# Progress in State Medicine

**Published:** 1902-06-28

**Authors:** 


### Progress in State Medicine.

### HOUSING OF THE WORKING CLASSES.

Yarrett[1] thinks that one of the most promising, or perhaps the most promising, of all sanitary reforms lies in the direction of house improvements for the lowest class of the population. Such a reform brings no profit or credit to any individual; there is no object in advertising it as in the case of private establishments for the cure of disease, and consequently it has taken a less firm hold upon the imagination of the public. In addition the provision of better houses can only be made by some sacrifice on the part of ratepayers of the present generation, and this fact, as soon as it comes to be appreciated, detracts greatly from the popularity of the subject. Larger houses are required, and it is useless providing new houses for the poor that do not afford much greater accommodation of space than the existing ones. There are too many little houses. Whilst there are laws to ameliorate most other insanitary conditions, there is no power to amend want of space. The limit of air-space agreed upon as that below which legal action shall be taken to abate overcrowding, viz., 300 cubic feet sleeping-space for an adult, and half that for children under 10 years of age, is much too low a limit. This limit is conventionally fixed, not by the requirements of sanitation, but by the driving necessity of taking cognisance of the innumerable tiny places which are in existence, and by the recognition of the fact that if the limit were raised to, say, 500 cubic feet sleeping-space, with a corresponding size for the day-rooms, a large part of the poor population would be turned out of house and home on account of overcrowding. A bedroom in a middle-class house has not uncommonly a cubic area of as much as 3,000 feet. This space in a poor house accommodates 10 adults without being overcrowded. It may be safely said that nothing that has been done in providing houses by sanitary authorities has had any considerable effect upon the great question of better housing for the poorest poor. The result of action under Part III. of the Housing of the Working Classes Act has been either to provide superior houses to let at a rental beyond the reach of the poorest and most needy, or to build as cheaply as possible small houses in which the space accommodation is far too little, and which are consequently quite unfit for occupation by poor families. These last houses letting at 1s. 6d. per week per room are not really cheap for the accommodation they afford. Before much progress can be expected the existing properties and property owners must be dealt with; many of the old houses must be put out of habitation without prejudice to the owners. The larger poorer families must be made to occupy the larger houses. The better class working family must not be allowed to move into the better houses at a lower rent. If Government passes a law permitting money to be borrowed by local sanitary authorities in such a way that the portion of the obligation of repayment shall fall upon our successors for the next two or three generations in the shape of continuing rates, it will be easier to build houses to let at low rates, and thus some portion of the difficulty will be solved. Fraser[2] is of opinion that useful lessons may be learnt by the consideration of the various elements which go to make up the existing rents of workmen's and labourers' houses. He selects as examples three types of two-apartment houses:—(1) Good-class artisans' houses let at £11 per annum, or 4s. 3d. per week; (2) "ticketed" house, let at £6 8s., or 2s. 5d. per week; and (3) model houses, as built by the Glasgow Workmen's Dwelling Company in Rottenrow, and let at £6 11s., or 2s. 6¼d. per week. The return in the first two instances represents, under favourable circumstances, 5 per cent. on the cost of the buildings and 3½ per cent. on the value of the land. In the third case the return is only 3½ per cent., but a provision is made against future repairs and depreciation. The company earns a better return from old disreputable properties, which they have converted into sound sanitary dwellings. In dealing with the question of interest on capital invested, a higher percentage must be obtained than the rate at which money can be borrowed, not only to provide for depreciation, but also to admit of a margin against the possibility of unlet houses and other risks. In the houses mentioned he states that it is cheaper for a workman or labourer to pay what is considered a high ground rent than to spend at least 1s. per week in car fares to bring him from his residence to his work. On going into the question of economising cost, it is found that a house can be produced 10 to 11 per cent. cheaper in a five-storey building than in a three-storey one. A house of large cubic capacity can be built relatively much cheaper than a small house. By economising at every turn and charging 4 per cent. on expenditure in building, houses might be produced ranging from 1s. 6d. for an apartment of 1,000 cubic feet, and up to 3s. per week for a fair-sized room and kitchen house of very plain finish. This would be upon ground costing 10s. per yard, and the rents would be exclusive of tenants' taxes. The committee[3] appointed at the Conference on the Housing Question held at Glasgow last September to consider how local authorities might obtain simpler and less costly machinery for putting in force the provisions of the Housing Acts, have resolved that the Government be approached with the view of getting a number of amendments made in the existing law. The proposals include the following:—(1) That in any improvement scheme one inquiry as to the expediency of the scheme should be sufficient for the sanction to borrow money, without the necessity of a further local inquiry, and that a provisional order approving the scheme should authorise the borrowing of the necessary moneys; (2) that provision should be made for the appointment of commissioners to hold local inquiries, and make orders to be confirmed by the Local Government Board, as is done under the Light Railways Act, empowering local authorities to put in force the compulsory power of purchase under the Land Clauses Acts; (3) that in fixing the compensation for land taken compulsorily there shall be no appeal from the award of the single arbitrator, except on a point of law; (4) that local authorities should have certain powers in making closing orders and of compulsory purchase and demolition of the property to which these relate; (5) that the Local Government Board in England and the Sheriff in Scotland should have power to authorise Local

Authorities to acquire lands compulsorily under the Lands Clauses Acts, without the necessity of an application to Parliament, and that there should be no allowance for compulsory purchase; and (6) that the term of repayment of loans should in all cases be extended to 60 years, and that as land is always an asset, the sinking fund should not be made applicable thereto. In the first volume of the Census Report[4] on the county of London it is shown that the total number of separate tenements in London in 1901 was 1,019,546, the increase in 10 years being equal to 8·7 per cent. The number of tenements containing five or more rooms was 347,516 an increase of 13·2 per cent., while the increase of those with fewer than five rooms did not exceed 6·6 per cent. This distinct improvement is further shown by the fact that the tenements of one room formed only 14·7, while in 1891, they formed 18·4 per 100 total dwellings of all kinds. The proportion of one-room tenements which were overcrowded was also less in 1901 than in 1891. Figures collated from this report show that 66 per cent. of the total families in London occupy tenements of fewer than five rooms, 15 per cent. occupied tenements of one room, 20 per cent. tenements of two rooms, 18 per cent. tenements of three, and 13 per cent. tenements of four rooms. With regard to the influence of size of tenement and degree of overcrowding on the death-rates, the metropolitan boroughs with the highest proportion of tenements containing fewer than five rooms, and with the highest proportion of overcrowding in these tenements, have the highest general death-rate and the highest death-rate from consumption. The low phthisical and general death-rate of Lewisham, Hampstead, and Wandsworth show the important favourable influence of what may be described as the more diluted housing of the people in these districts. Attention is drawn to the fact that there are fashionable residential districts in which the overcrowding is so widespread and serious as to call for large measures of housing in these districts. The report[5] of the Housing of the Working Classes Committee recently presented to the London County Council, states that in 1891 there were 831,668 persons living under overcrowded conditions in 145,844 tenements of less than five rooms. This gives an average of 2·99 persons per room. In 1901 there were 726,096 persons living under overcrowded conditions in 124,773 tenements of less than five rooms. This gives an average of 2·88 persons per room. Both the amount and density of overcrowding in such tenements appear to have been reduced since 1891, except in the case of the Borough of Stepney. With regard to tenements of more than five rooms, in 1891 there were 1,889,475 persons living in 308,918 such tenements, thus giving an average of 6·11 persons per tenement. In 1901 there were 2,086,702 persons living in 347,516 such tenements, thus giving an average of 6·00 persons per tenement. There appears, therefore, to have been an increase in the number of tenements of more than five rooms, and an increase in the number of persons occupying such tenements, but a decrease of 0·11 in the average number of persons per tenement. This again points to a decrease in the prevalence of overcrowding since 1891.

[1] Sanitary Rec., April 17, 1902. [2] Public Health Engineer, March 29, 1902. [3] Sanitary Rec., March 27, 1902. [4] Brit. Med. Jour., March 8, 1902. [5] Brit. Med. Jour., March 22, 1902.

